# Comparison of Two Different Canine Anti-IgG Antibodies for Assessment of Oligoclonal Bands in Cerebrospinal Fluid and Serum of Dogs *via* Isoelectric Focusing Followed by an Immunoblot

**DOI:** 10.3389/fvets.2022.873456

**Published:** 2022-07-05

**Authors:** Julia K. Prümmer, Veronika M. Stein, Eliane Marti, Mario Ziegler, Andreas Lutterotti, Ilijas Jelcic, Frank Steffen, Thorsten Buch, Arianna Maiolini

**Affiliations:** ^1^Clinical Neurology, Vetsuisse Faculty, University of Bern, Bern, Switzerland; ^2^Division of Neurological Sciences – Clinical Immunology, Vetsuisse Faculty, University of Bern, Bern, Switzerland; ^3^Department of Neurology, University of Zurich, Zurich, Switzerland; ^4^Clinical Neurology, Vetsuisse Faculty, University of Zurich, Zurich, Switzerland; ^5^Institute of Laboratory Animal Science, University of Zurich, Zurich, Switzerland

**Keywords:** canine (dog), cerebrospinal fluid (CSF), inflammation, immunoblot, isoelectric focusing (IEF)

## Abstract

Isoelectric focusing followed by immunoblotting is a method routinely used in human medicine to assess the presence of oligoclonal bands (OCBs) in cerebrospinal fluid (CSF) and serum. The detection of OCBs is a valuable diagnostic test, especially important in patients with the suspicion of multiple sclerosis (MS), in which at least two OCBs are found in the CSF not present in paired serum samples in up to 95% of patients. So far, presence of OCBs in CSF and serum of dogs has only been investigated in a small cohort of dogs diagnosed with degenerative myelopathy and healthy dogs. The main objective of the current study was to describe the method used for OCB detection and compare two different canine anti-IgG antibodies: a canine rabbit-anti-IgG antibody (Jackson ImmunoResearch) vs. a canine goat-anti-IgG antibody (Bio-Rad). The method was performed according to the instructions of the commercial kit used. The canine goat-anti-IgG antibody showed a better performance than the canine rabbit-anti-IgG antibody. The availability of the technique of OCB detection in the dog paves the way for further studies, especially in the field of inflammatory diseases of the canine central nervous system, and comparison between specific human and canine diseases.

## Introduction

The analysis of cerebrospinal fluid (CSF) is an important diagnostic test for various neurological diseases in both human and veterinary patients. Besides the standard macroscopic, biochemical, and cytological analyses, the assessment of IgG oligoclonal bands (OCBs) is routinely performed in human medicine. B cells and plasma cells involved in an inflammatory process are responsible for the production of oligoclonal immunoglobulin within the CNS; the term oligoclonal means the immunoglobulin is derived from few antibody clones (1). OCBs that are present in CSF but not in the paired serum sample are representing a local humoral response in the CNS (2). They are found in various infectious, autoimmune, and inflammatory diseases in human medicine (1, 3), with multiple sclerosis (MS) being the disease with the highest incidence of OCBs. Although there is a lack of understanding of the precise role of OCBs in MS, their presence is tightly coupled with the disease: up to 95% of human MS patients show two or more OCBs exclusively in the CSF, which are absent in the paired serum sample (2). OCB analysis is the most sensitive method for qualitative assessment of intrathecal IgG synthesis (4). Different methods for their analysis have been used and investigated; isoelectric focusing (IEF) on agarose gel followed by immunoblotting for IgG using paired CSF and serum samples is most widely used in clinical routine for the detection of OCBs (2, 5–7). Polyacrylamide gel electrophoresis and IEF combined with silver staining of proteins may be more sensitive, but is used less commonly in clinical routine since the method is more time-consuming, costly and dependent on elaborate expertise (1). According to the 2017 McDonald criteria for the diagnosis of MS, the presence of CSF-specific OCB may substitute for the requirement for demonstration of dissemination of time in relapsing-remitting MS and is one of three additional criteria for the diagnosis of primary progressive MS (2, 6, 8, 9). In veterinary medicine, OCBs in CSF and serum so far have only been investigated in six German Shepherd dogs diagnosed with degenerative myelopathy using a modified IEF and immunofixation method (10).

The purpose of the present study was to describe a modified method for OCB detection in dogs as well as to compare two different canine anti-IgG antibodies for detection of OCBs in CSF and serum samples of dogs. For this purpose, isoelectric focusing followed by immunoblotting used in human medicine was investigated in a clinical laboratory specialized in human CSF diagnostics.

## Materials and Methods

The experimental procedures used in this study were approved by the Ethical Committee of the Veterinary Service, Cantone of Bern (BE121/2020).

### Inclusion of Dogs

Dogs included in the study were presented for diagnostic purposes to the Small Animal Clinic, Division of Clinical Neurology, Vetsuisse Faculty Bern, as well as to the Neurology Department, Clinic of Small Animal Surgery, Vetsuisse Faculty Zurich and were diagnosed with different neurological diseases [meningoencephalitis of unknown origin (MUO) *n* = 8, idiopathic epilepsy (IE) *n* = 2, intracranial neoplasia (IN) *n* = 1, intervertebral disc herniation (IVDH) *n* = 2, steroid-responsive meningitis-arteritis (SRMA) *n* = 3, eosinophilic meningoencephalitis *n* = 1], as well as three dogs that did not receive a final diagnosis. The medical records including signalment of all dogs were available. Moreover, all dogs included received a complete diagnostic work-up with physical and neurological examination, as well as CSF analysis. The further diagnostic work-up differed according to the suspected underlying disease and included one or more of the following examinations: hematology, biochemistry, infectious disease testing, urine analysis, thorax and/or abdominal radiographs, MRI of the brain and/or spinal cord. The samples used for this brief research report were investigated to describe the method and compare two different canine anti-IgG antibodies. The description of CSF-specific OCBs in neurological and non-neurological diseases and a statistical comparison among different groups are not objects of this communication.

### Collection of CSF and Serum Samples of Dogs

Paired CSF and serum samples were collected from all dogs after the MRI examination of the brain and/or spinal cord for the underlying disease in general anesthesia. CSF was obtained by atlanto-occipital puncture in lateral recumbency, harvested into plain polypropylene tubes, and clinical standard examinations were performed within 30 min. Standard clinical CSF analysis included total protein, white blood cell count and cell differentiation. Blood samples mostly were taken at the same time or within 24 h by puncture of a peripheral vein (e.g., V. saphena lateralis or V. cephalica antebrachii). Serum samples were centrifuged (10 min, 3000 × *g*, at 20°C) and CSF and serum samples frozen at −80°C within 1 h after collection. Samples were thawed prior to the measurement of the IgG concentration and further processing for OCB detection.

### Measurement of IgG Concentration

The IgG concentration of all samples had to be determined, as CSF and serum samples needed to be adjusted to the same IgG amount for the later assessment of OCBs and comparability of both samples. A canine IgG ELISA kit (Abcam, Cambridge, United Kingdom) was used according to the manufacturer‘s protocol. A pilot study was performed to determine suitable serum and CSF dilutions to be used in the ELISA, using samples from two dogs. Ten-fold serum dilutions ranging from 1:1,000 to 1:1,000,000 were made and CSF was tested undiluted and in serial dilutions up to 1:10,000. Values were within the standard curve using a serum dilution of 1:100,000 and CSF dilution of 1:1,000. These dilutions were used for all following IgG measurements in CSF and serum. Moreover, serum and CSF dilutions of the two dogs initially used to establish the ideal dilution were used as controls for all further ELISA plates measured. The median IgG concentration in CSF and serum was 137.66 mg/L and 23.32 g/L with a mean of 352.52 mg/L and 24.46 g/L, respectively.

### Assessment of OCBs in CSF and Serum of Dogs *via* Isoelectric Focusing Followed by an Immunoblot

IEF and subsequent immunoblot were performed according to the instructions of the human kit provided (SEBIA Swiss GmbH, Wollerau, Switzerland).

#### Preparation of the Agarose Gel Plate and Running of the IEF

**Figure 1:**
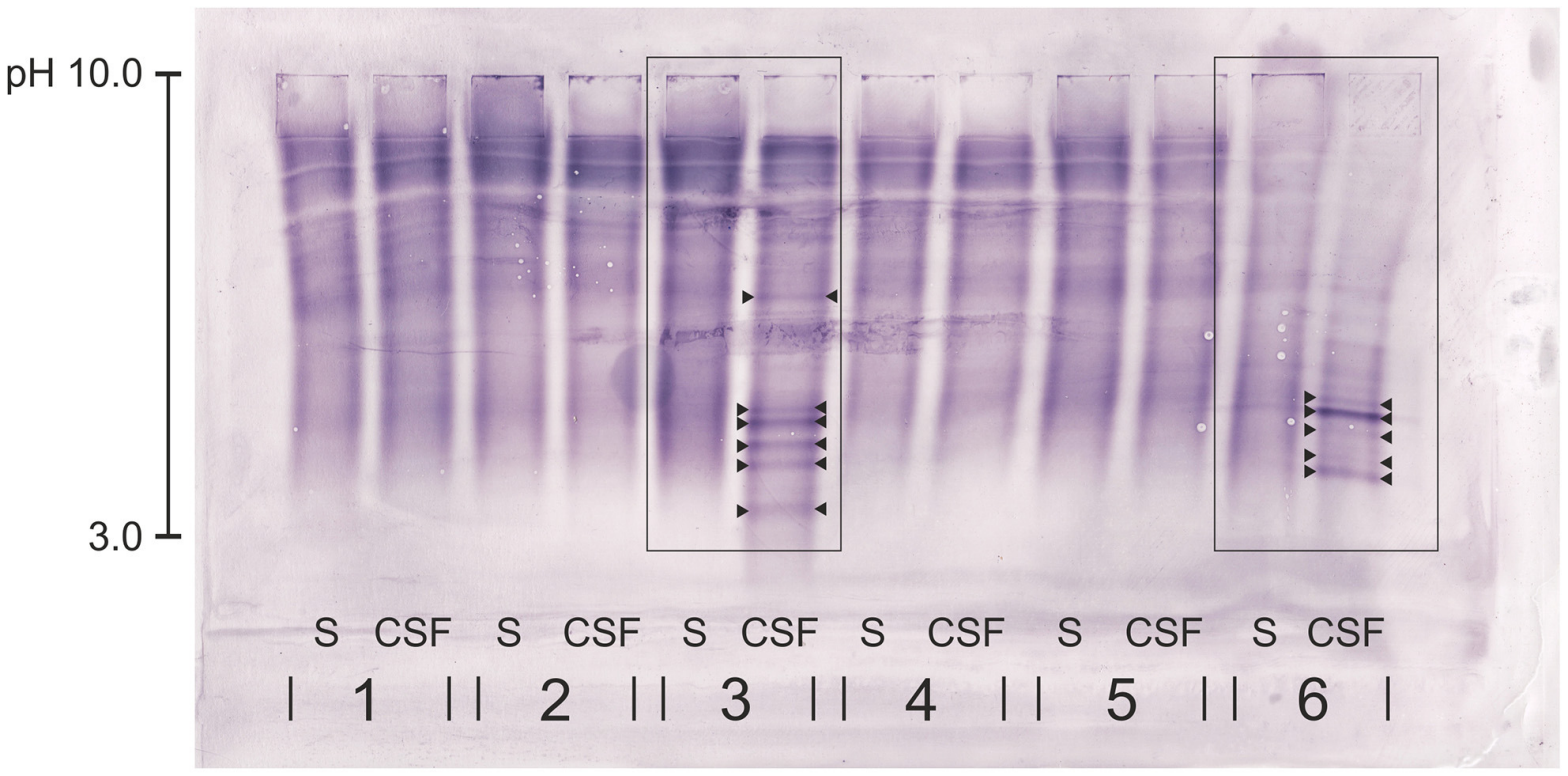
Immunoblot of five canine serum and CSF samples (1-5) and the human samples (6). Canine rabbit-anti-IgG antibody was used. Dog no. 3—with chronic intervertebral disk protrusions—presents with clear CSF-specific OCBs: more than five OCBs are detected in the CSF, but not in the corresponding serum sample. The human sample (6) serving as positive control shows clear CSF-specific OCBs. OCBs are indicated by arrowheads. S, serum; CSF, cerebrospinal fluid.

Samples were thawed at room temperature before their analysis and diluted with *Aqua ad iniectabilia* to reach equal IgG concentrations of 25 mg/mL. The diluted serum and CSF samples were vortexed and 7 μL per sample were pipetted into the wells of the agarose gel (1%). In case the original IgG concentration was <25 mg/mL, the total amount was increased accordingly to reach an equal amount of IgG. As a standard canine positive control was not available due to restricted CSF and serum sample volumes in canine patients, a human CSF and serum sample were used as controls (Figure 1). The presence of CSF-specific OCBs in these samples was confirmed using a human anti-IgG antibody. Samples were allowed to absorb in the agarose gel for 10 min, and the contact strips were placed on the correct position on the gel. IEF was performed in a semi-automated analyzer “EasyFix Interlab G26” (Apteq AG, Cham, Switzerland) according to the manufacturer's instructions. The purpose of the IEF is to fractionate the proteins in the CSF and serum samples on agarose gel according to their isoelectric point (anode pH 3.0, cathode pH 10.0, current 70 mA, voltage 505 V, and duration of 75 min).

#### Westernblot

After completion of the IEF, the gel was removed from the frame and placed onto a soft paper tissue. Both contact strips were removed and the gel placed centrally on an aluminum plate. One blotting membrane (nitrocellulose membrane) was immerged in a plastic container with *Aqua destillata* (20 mL), quickly dried between two soft paper tissues and then placed onto the gel in order to remove excess proteins and fluid. The lower right corner of a second blotting membrane was removed, and the membrane placed into a plastic container with 20 mL *Aqua destillata* and 1.6 mL methanol 100% (helps to transfer the proteins onto the membrane). The membrane was then placed onto the gel, carefully smoothed with gloves until no air bubbles or irregularities were visible and covered with the smooth surface of a blotter G paper. A second aluminum plate was placed on top of the membrane and two weights (each 1 kg) applied on top. The membrane was blotted for 30 min.

#### Immunoblot

After blotting, the membrane was placed with the blotting side upwards into a plastic container with 20 mL of Binding agent 1 and incubated for 30 min on a shaker. The blotting membrane was then washed twice in 0.9% saline solution for 2 min and placed into a plastic container with 20 mL of Binding agent 2 (=dilution of Binding agent 1: 1:10). Two canine anti-IgG antibodies were used for the measurements. The first one was a canine rabbit-anti-IgG antibody (Jackson ImmunoResearch Laboratories, Ely, United Kingdom; H+L, purified, polyclonal, Alkaline Phosphatase conjugated; product code 304-055-033), of which 80 μL in a concentration of 0.6 mg/mL were used. This antibody was chosen based on the material used in the only clinical study on canine OCBs (10). The second antibody was a canine goat-anti-IgG antibody (Bio-Rad, Cressier, Switzerland; H+L, purified, polyclonal, Alkaline Phosphatase conjugated, product code AAI50A), of which 50 μL in a concentration of 1.0 mg/mL were used. The latter was chosen as it presented with similar characteristics as the first one used as well as the human anti-IgG antibody (polyclonal, Alkaline Phosphatase conjugated). As the exact concentration of the human antibody provided by the kit was not known (given range by the company from 0.8 to 2.5 mg/mL, 20 μL per blot was used), we decided to apply four times the amount of the antibody used for human samples, which is equivalent to the upper range of the concentration of the human antibody. The remaining antibody was aliquoted and diluted with glycerol (American Chemical Society; ACS grade or better). The aliquots were stored at −20°C. The blotting membrane was then incubated with Binding agent 2 and the respective anti-dog IgG antibody for 1 h and the membrane washed again twice with 0.9% saline solution for 3 and 2 min and subsequently twice with *Aqua destillata* for 3 and 2 min, respectively. The “final reaction solution” was prepared by adding 4 mL of the CSF Substrate (Indoxylphosphate in alkaline buffer) to the NBT CSF (Nitroblue tetrazolium CSF) and shaking it for 10 min. The blotting membrane was then transferred into a small plastic container and the whole solution was placed on top of the membrane. The membrane was incubated on a plate shaker for 5–7 min; depending on the time necessary until the bands of the control samples were nicely visible. Following the incubation, the membrane was washed again twice for 5 min first with 0.9% saline solution and then *Aqua destillata* and dried using soft tissues and a weight.

#### Evaluation of the Immunoblots

All blots were evaluated by three blinded experienced examiners (AL, MZ, and EdA) working in the human laboratory specialized for OCB detection and using the method on a daily basis for more than 5 years. The presence of the positive control, overall correct running of the samples on the gel, and correct blotting were evaluated by all examiners. The result was classified as sufficient for interpretation, if the OCB pattern of the human control sample assessed with the human anti-IgG antibody was reflected by the result of the samples assessed with the canine anti-IgG antibody. Additionally, at least two out of three examiners needed to be able to read and evaluate the samples of a patient. The final evaluation of the OCB result was performed using an adapted scheme of the current guidelines in human medicine describing five patterns: pattern 1 = no OCB in CSF and serum; pattern 2 = CSF-specific OCBs, i.e. presence of OCBs in CSF, not in serum; pattern 3 = CSF-specific OCBs with additional, identical bands in CSF and serum; pattern 4 = identical OCBs in CSF and serum; and pattern 5 = monoclonal bands in CSF and serum (7). CSF-specific OCBs—equivalent with the previously described pattern 2 or 3—were defined as the presence of at least two additional bands uniquely present in the CSF, but not in the corresponding serum (Figure 1). For the purpose of the presented study, the focus lies on the presence or absence of CSF-specific OCBs, rather than single specific patterns or exact number of OCBs.

## Results

**Figure 2:**
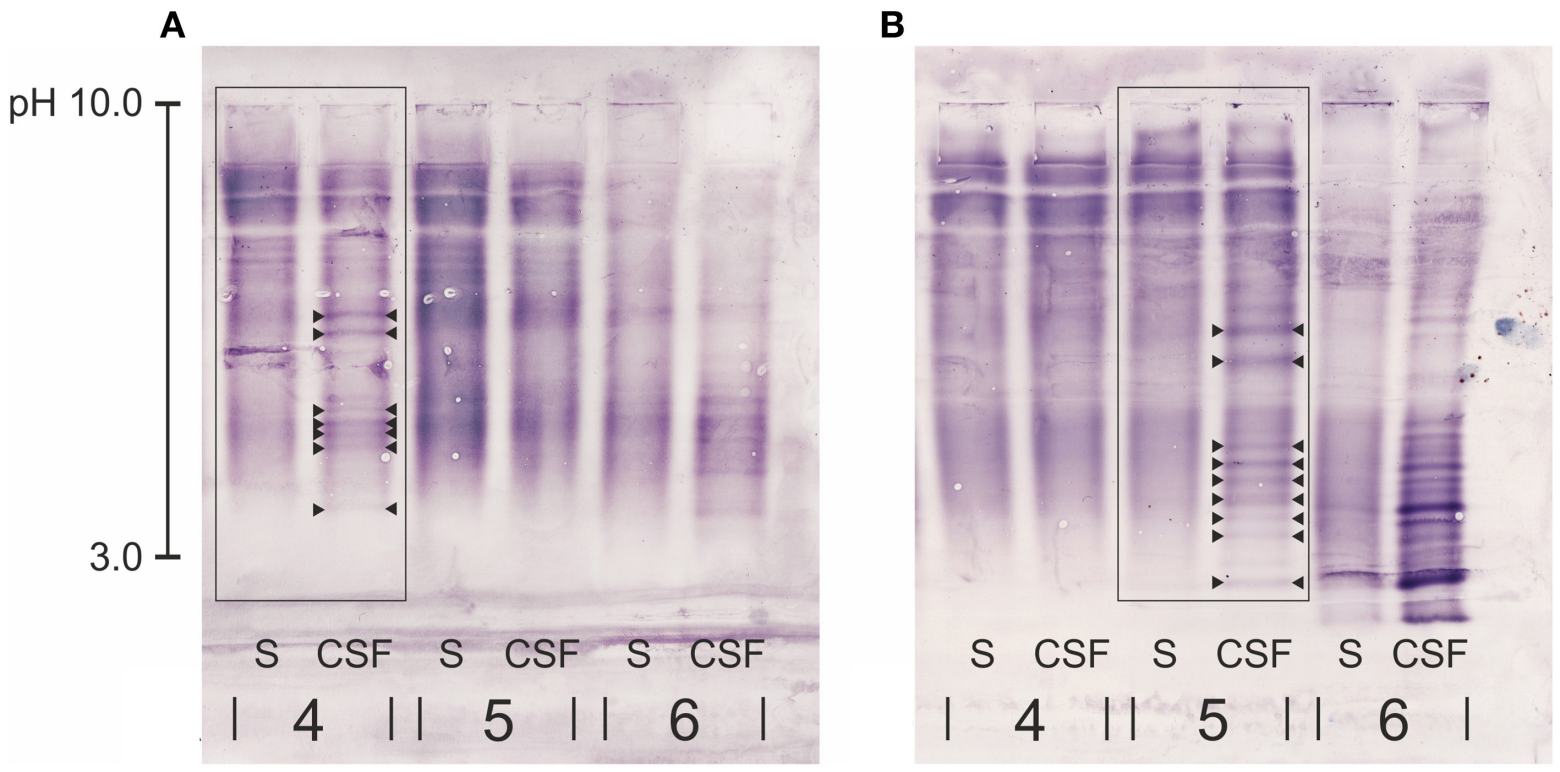
Close-up of part of two immunoblots of each two canine serum and CSF samples (4, 5) and the human samples serving as positive control (6) for comparison of two different anti-dog IgG antibodies: **(A)** canine rabbit-anti IgG antibody, **(B)** canine goat-anti-IgG antibody. Patient no. 4 in **(A)** is the same patient as no. 5 in **(B)** diagnosed with intracranial neoplasia. Both immunoblots of this patient show the same pattern of CSF-specific OCBs, however the canine goat-anti- IgG in **(B)** produced a less pinkish background and OCBs were more clearly visible. OCBs are indicated by arrowheads. S, serum; CSF, cerebrospinal fluid.

CSF and serum sample of 20 dogs were examined for IgG concentration and presence of OCBs. In those 20 paired samples, the performance of two different canine anti-IgG antibodies was evaluated. Results of 16/20 (80%) dogs examined using the canine rabbit-anti-IgG antibody compared to 20/20 (100%) of dogs assessed with the canine goat-anti- IgG antibody were sufficient for evaluation of the different patterns described above. Overall, there was an excellent agreement of pattern recognition between all three examiners (CSF-specific OCBs yes or no). On contrary, there was a disagreement concerning the number of visible OCBs in 8/16 cases (50%) of dogs evaluated with the canine rabbit-anti-IgG antibody and in 12/20 cases (40%) evaluated with the canine goat-anti-IgG antibody. In these cases, the blot was evaluated again by the most experienced examiner (AL) in order to reach a consensus. Both canine anti- IgG antibodies showed cross-reactivity with human serum and CSF samples; a feature that was used to include a human sample with clear visible CSF-specific OCBs as positive control. Overall, when comparing the performance of the canine goat-anti-IgG antibody and the canine rabbit-anti-IgG antibody, both consistently lead to the same conclusion concerning the presence or absence of CSF-specific OCBs in 16/16 (100%) cases. In comparison with the canine rabbit-anti-IgG antibody, the canine goat-anti IgG antibody caused significantly less pinkish background, which facilitated detection of faint bands (Figures 2A,B) and made the evaluation in general easier, which is reflected by the higher agreement between the examiners when evaluating the quantity of bands (as mentioned above). Consequently, in 5/16 (31%) patients, more OCBs in CSF and/or serum became visible (Table 1). However, this did not change the conclusion of the qualitative assessment (presence vs. absence) of CSF-specific OCBs.

**Table 1:** Comparison of two different canine anti-IgG antibodies.

**Dog Nr**.	**Jackson ImmunoResearch^a^** **CSF-specific OCBs y/n**	**Bio-Rad^b^** **CSF-specific OCBs y/n**	**Jackson ImmunoResearch^a^** **Number of OCBs in Serum/CSF**	**Bio-Rad^b^** **Number of OCBs in Serum/CSF**
1	n	n	0/0	0/0
2	–	y	–	0/>5
3	y	y	0/2	0/2
4	–	n	–	0/0
5	n	n	0/0	0/0
6	–	n	–	0/0
7	y	y	**0/3**	**0/5**
8	–	y	–	0/4
12	n	n	0/0	0/0
23	y	y	0/>5	0/>5
39	y	y	0/4	0/4
49	y	y	**0/3**	**1/5**
58	y	y	0/4	0/4
67	n	n	0/0	0/0
70	y	y	0/>5	0/>5
78	y	y	**1/3**	**0/>5**
90	n	n	0/0	0/0
102	y	y	**2/5**	**1/>5**
106	n	n	**0/0**	**0/1**
107	y	y	0/>5	0/>5

*Overall, the result of CSF-specific OCBs yes (y) or no (n) did not differ between both antibodies. Consistent results regarding number of OCBs were found in 11/16 patients. In 5/16 patients, the canine goat-anti-IgG antibody showed more OCBs (numbers in bold). CSF, cerebrospinal fluid; OCB, oligoclonal bands*.

a *Jackson ImmunoResearch Laboratories, Ely, United Kingdom*.

b *Bio-Rad, Cressier, Switzerland*.

## Discussion

The assessment of OCBs using a commercially available isoelectric focusing protocol followed by an immunoblot is routinely used in human medicine, especially during the diagnostic work-up of a presumed MS. In contrast, the diagnostic value of assessing OCBs in various diseases in dogs has not been investigated. Therefore, the present study paves the way to use the method of OCB detection in a larger clinical trial in dogs for the future. For the assessment, dogs with a variety of different clinical diagnoses were included; inclusion of healthy dogs was limited by ethical restrictions. Kamishina et al. assessed the prevalence of OCBs in CSF and serum obtained post mortem in six clinically healthy dogs; none of those presented CSF-specific OCBs (10). Two different canine anti-IgG antibodies were compared, with the canine goat-anti-IgG antibody overall presenting a better performance. There is not much information about the method and performance of the antibodies in human medicine; it is estimated that 10–20% of samples need to be repeated (personal communication MZ). This estimation is comparable with the performance of the rabbit canine anti-IgG antibody used in the present study, where the analysis was not sufficient for evaluation in 20% of the canine samples. In contrast, all the samples evaluated using the goat canine anti-IgG antibody were successfully evaluated.

The current study presents some limitations. Firstly, only a small amount of canine samples has been evaluated and results might differ in a larger cohort. Secondly, the three examiners did not agree on the exact number of OCBs found in each sample in 50 and 40% of the dogs, when evaluated with the rabbit canine anti-IgG antibody or the goat canine anti-IgG antibody, respectively. It is important to remember that the assessment of OCBs in serum and CSF is a qualitative, not quantitative assessment. Therefore, the main conclusion is the presence or absence of CSF-specific OCBs, per definition two or more OCBs uniquely present in the CSF sample, independently from the exact number of OCBs. A third limitation is linked to the intrinsic nature of the method, being the evaluation of OCBs a subjective assessment, which can lead to both false-positive and false-negative results. Finally, the antibodies used recognize both heavy and light chains of the immunoglobulin, therefore we cannot fully exclude that also additional immunoglobulin classes other than IgG have been detected.

In summary, both antibodies enabled the interpretation for the presence or absence of CSF-specific OCBs in the canine samples; overall, the canine goat-anti-IgG antibody showed a better performance than the canine rabbit-anti-IgG antibody. Besides MS, OCBs have been detected in many different diseases in humans such as other inflammatory conditions, infectious or neoplastic diseases (3). In contrast, OCBs in veterinary medicine have been investigated only in dogs with degenerative myelopathy (10). Establishing the method for OCB detection in dogs allows its use in various neurological diseases and can potentially assist in the diagnosis of canine immune-mediated meningoencephalomyelitis. The authors are currently investigating the prevalence of OCBs in CSF and serum in a larger cohort of dogs with different neurological diseases.

The availability of the methodology for OCB detection in dogs paves the way for further insights concerning the pathophysiology of inflammatory diseases and could support clinical diagnostics in these diseases.

## Data Availability Statement

The raw data supporting the conclusions of this article will be made available by the authors, without undue reservation.

## Ethics Statement

The animal study was reviewed and approved by Ethical Committee of the Veterinary Service, Cantone of Bern (BE121/2020). Written informed consent was obtained from the owners for the participation of their animals in this study.

## Author Contributions

JP: conceptualization, laboratory analyses, writing—original draft preparation, and writing—review and editing. VS: conceptualization, writing and editing, supervision, and resources. MZ: introduction and helped with laboratory work, methodology, evaluation, and interpretation of laboratory results. AL and IJ: conceptualization, methodology, evaluation, and interpretation of laboratory results. FS and TB: conceptualization and writing—review and editing. AM: conceptualization, writing and editing, and supervision. All authors read and approved the final version of the manuscript.

## Funding

This work was supported by the Board of Specialization of the Vetsuisse Faculty of Veterinary Medicine, Bern. The funding source was not involved in study design, laboratory work, writing of the article, or decision to submit the article for publication. Open access funding was provided by the University of Bern.

## Conflict of Interest

The authors declare that the research was conducted in the absence of any commercial or financial relationships that could be construed as a potential conflict of interest.

## Publisher's Note

All claims expressed in this article are solely those of the authors and do not necessarily represent those of their affiliated organizations, or those of the publisher, the editors and the reviewers. Any product that may be evaluated in this article, or claim that may be made by its manufacturer, is not guaranteed or endorsed by the publisher.
