# Erratum: Comparison of two different canine anti-IgG antibodies for assessment of oligoclonal bands in cerebrospinal fluid and serum of dogs *via* isoelectric focusing followed by an immunoblot

**DOI:** 10.3389/fvets.2023.1182142

**Published:** 2023-03-22

**Authors:** Frontiers Production Office

**Affiliations:** Frontiers Media SA, Lausanne, Switzerland

**Keywords:** canine (dog), cerebrospinal fluid (CSF), inflammation, immunoblot, isoelectric focusing (IEF)

An omission to the funding section of the original article was made in error. The following sentence has been added: “Open access funding was provided by the University of Bern.”

The original article has been updated.

